# Low-Dissipation Thermosets Derived from Oligo(2,6-Dimethyl Phenylene Oxide)-Containing Benzoxazines

**DOI:** 10.3390/polym10040411

**Published:** 2018-04-07

**Authors:** Chien-Han Chen, Kuan-Wei Lee, Ching-Hsuan Lin, Tzong-Yuan Juang

**Affiliations:** 1 Department of Chemical Engineering, National Chung Hsing University, Taichung 402, Taiwan; jain6792002@gmail.com (C.-H.C.); tgdyt547165@gmail.com (K.-W.L.); 2 Department of Cosmeceutics, China Medical University, Taichung 404, Taiwan; tyjuang@mail.cmu.edu.tw

**Keywords:** benzoxazine, low-dissipation, poly(phenyl oxide)

## Abstract

Poly(2,6-dimethyl phenyl oxide) (PPO) is known for its low dissipation factor. To achieve insulating materials with low dissipation factors for high-frequency communication applications, a telechelic oligomer-type benzoxazine (P-APPO) and a main-chain type benzoxazine polymer (BPA-APPO) were prepared from an amine end-capped oligo (2,6-dimethyl phenylene oxide) (APPO). The APPO was prepared from a nucleophilic substitution of a phenol-end capped oligo (2,6-dimethyl phenylene oxide) (a commercial product, SA 90) with fluoronitrobenzene, and followed by catalytic hydrogenation. After self-curing or curing with a dicyclopentadiene-phenol epoxy (HP 7200), thermosets with high-*T*_g_ and low-dissipation factor can be achieved. Furthermore, the resulting epoxy thermosets show better thermal and dielectric properties than those of epoxy thermoset cured from its precursor SA90, demonstrating it is a successful modification in simultaneously enhancing the thermal and dielectric properties.

## 1. Introduction

The dielectric loss (*L*) is proportional to the product of square root of dielectric constant (*D*_k_^1/2^), frequency, and dissipation factor (*D*_f_). Therefore, the dielectric loss will be more obvious for application in high-frequency communication. In addition, *D*_f_ will be more important than *D*_k_ since *D*_f_ is directly proportional the *L*, while there is only a square root relationship between *D*_k_ and *L*. 

Polybenzoxazines feature for special characteristics such as a moderate-to-high thermal property [1,2,3,4,5,6,7,8,9,10,11,12,13,14,15,16,17,18], moderate electrical property [19], low water absorption [20], and low surface energy [21]. Low-dielectric benzoxazines have been reported (1) by Chang et al. through Mannich condensation of bisphenol F/4-(trifluoromethyl)aniline/formaldehyde [22]; (2) by Liu et al. through incorporation of methyl methacrylate-POSS (MMA-POSS) into the network of bis(3-furfuryl-3,4-dihydro-2*H*-1,3-benzoxazinyl)isopropane (BPA-FBz), which has furfuryl moiety that can react with methyl methacrylate through the Diels-Alder reaction [23]; (3) by Liu et al. through the incorporation of a benzobisoxazole structure [24,25]; (4) by Alagar et al. through the incorporation of silica (SBA-15) [26]; (5) by Ishida et al. from a highly fluorinated diamine and bisphenol F [27]; and (6) by Lin et al. from fluorinated aromatic diamine [28]. However, to the best of our knowledge, no data show that thermosets of these benzoxazines can reach the requirement of low dissipation factor below 0.005 U for application in high-frequency communication. 

Poly(2,6-dimethyl phenylene oxide) (PPO) is a hydrophobic polymer developed by Hay et al. through the aromatic oxidative polymerization [29,30]. PPO is known for its low dissipation factor. Therefore, PPO is a building block to prepare low-dissipation materials. Thermosetting low-dielectric PPOs have been reported by Ueda et al. [31,32]. Low-molecular-weight telechelic PPOs have been developed by White et al. [33,34,35], Jayakannan et al. [36], and Percec et al. [37], Telechelic PPO oligomers with phenyl methacrylate end group has been commercialized by SABIC in the name of NORYL™ SA9000 resin. Vinyl benzyl ether-terminated PPO has been commercialized by Mitsubishi Gas Chemical in the name of OPE-2st.

**Figure 1:**
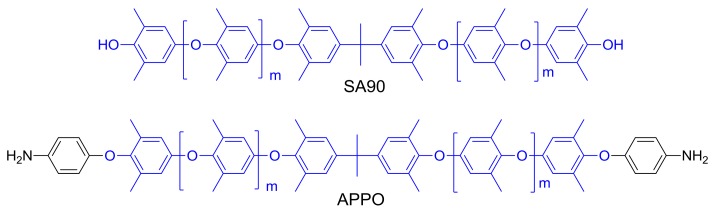
Structures SA90 and APPO. Note that there are no free ortho for SA90.

Difunctional benzoxazines are generally prepared by Mannich condensation of biphenol/monoamine/formaldehyde. Figure 1 shows the structure of SA90, a phenolic hydroxyl-end capped oligo (2,6-dimethyl phenylene oxide), commercialized by SABIC. The end group of SA90 is the 2,6-dimethyl phenolic OH, which does not contain free ortho, so it cannot carry out the Mannich condensation with primary amine and formaldehyde directly. In addition to the Mannich condensation of biphenol/monoamine/formaldehyde, difunctional benzoxazines can also be prepared from the Mannich condensation of diamine/monophenol/formaldehyde. Therefore, the only way to prepare PPO-containing benzoxazines is transforming the phenolic hydroxyls to amino groups. In this work, an amine end-capped oligo (2,6-dimethyl phenylene oxide) (APPO, Figure 1) was prepared from a nucleophilic substitution of SA90 and fluoronitrobenzene, and followed by catalytic hydrogenation. 

With APPO in hand, a telechelic oligomer-type benzoxazine from phenol, APPO, and formaldehyde was prepared by two approaches. The first approach is through a two-pot procedure to obtain the benzoxazine (P-APPO-2) [38]. The second approach is a one-pot Mannich condensation of phenol/APPO/formaldehyde to obtain the benzoxazine (P-APPO-1). A main-chain type benzoxazine polymer (BPA-APPO) was also prepared from the Mannich condensation of bisphenol A/APPO/formaldehyde using the mixed solvent approach [39]. Experimental data show that thermosets with very low dissipation factors can be achieved. Detailed synthesis, characterization, and the property are provided in this work.

## 2. Experimental Section

### 2.1. Materials

NORYL^TM^ SA90 (the weight average molecular weight 1700 g/mol, and the number average molecular weight 1600 g/mol) was purchased from SABIC (Riyadh, Saudi Arabia). Dicyclopentadiene novolac epoxy (HP7200) with an epoxy equivalent per weight (EEW) of 250 g/eq was kindly supplied by Dainippon Ink and Chemicals Corporation (Tokyo, Japan). Potassium carbonate (from Showa, Tokyo, Japan), 4-fluoro-1-nitrobenzene (from Alfa, Heysham, England), bisphenol A (from Acros, NJ, USA), paraformaldehyde (from TCI, Tokyo, Japan), 2-hydroxybenzaldehyde (from Showa), sodium borohydride (from Alfa), phenol (from Showa), palladium on carbon 10% (from Acros), and 4-dimethylaminopyrdine (DMAP, from Alfa) were purchased from various commercial sources and used without further purification. *N*-methylpyrrolidone (NMP; HPLC grade from Showa) and *N*,*N*-dimethyl acetamide (DMAc, HPLC grade from Showa) were purified by distillation under reduced pressure over calcium hydride (from Acros), and stored over molecular sieves. The other solvents (HPLC grade) were purchased from various commercial sources and used without further purification.

### 2.2. Characterization

Thermogravimetric analysis (TGA) was performed with a Perkin-Elmer Pyris1 (Perkin-Elmer, Shelton, CT, USA) at a heating rate of 20 °C/min in an atmosphere of nitrogen or air atmosphere. Dynamic mechanical analysis (DMA) was performed using a Perkin-Elmer Pyris Diamond DMA (Perkin-Elmer, Shelton, CT, USA) with a sample size of 5.0 cm × 1.0 cm × 0.2 cm. The storage modulus *E*’ and tan δ were determined as the sample was subjected to the temperature scan mode at a programmed heating rate of 5 °C/min at a frequency of 1 Hz. The test was performed using a bending mode with an amplitude of 5 μm. *T*_g_ value is determined from the peak temperature of the tan δ curve. Dielectric properties were performed with Agilent E4991A (Agilent, Santa Clara, CA, USA) at 1 GHz at 25 °C. The sample size is 2 × 2 cm^2^ with thickness at around 300 μm. Thermal mechanical analysis (TMA) was performed by an SII TMA/SS6100 (Seiko, Torrance, CA, US) at a heating rate of 5 °C/min. The coefficient of thermal expansion (CTE) was determined in the range of 50 to 150 °C. *T*_g_ value is determined from onset temperature. NMR measurements were performed using a Varian Inova 600 NMR (Agilent, Palo Alto, CA, USA) in DMSO-*d*_6_, and the chemical shift was calibrated by setting the chemical shift of DMSO-*d*_6_ at 2.49 ppm. IR spectra were obtained from at least 32 scans in the standard wavenumber range of 667–4000 cm^−1^ using a Perkin-Elmer RX1 infrared spectrophotometer (Perkin-Elmer, Shelton, CT, USA). Gel permeation chromatography (GPC) was performed by a Hitachi L2400 (Hitachi, NY, USA) in Tetrahydrofuran (THF), using polystyrene as a standard. 

### 2.3. Synthesis of Nitro End-Capped Oligo (2,6-Dimethyl Phenylene Oxide) (NPPO)

**Scheme 1:**
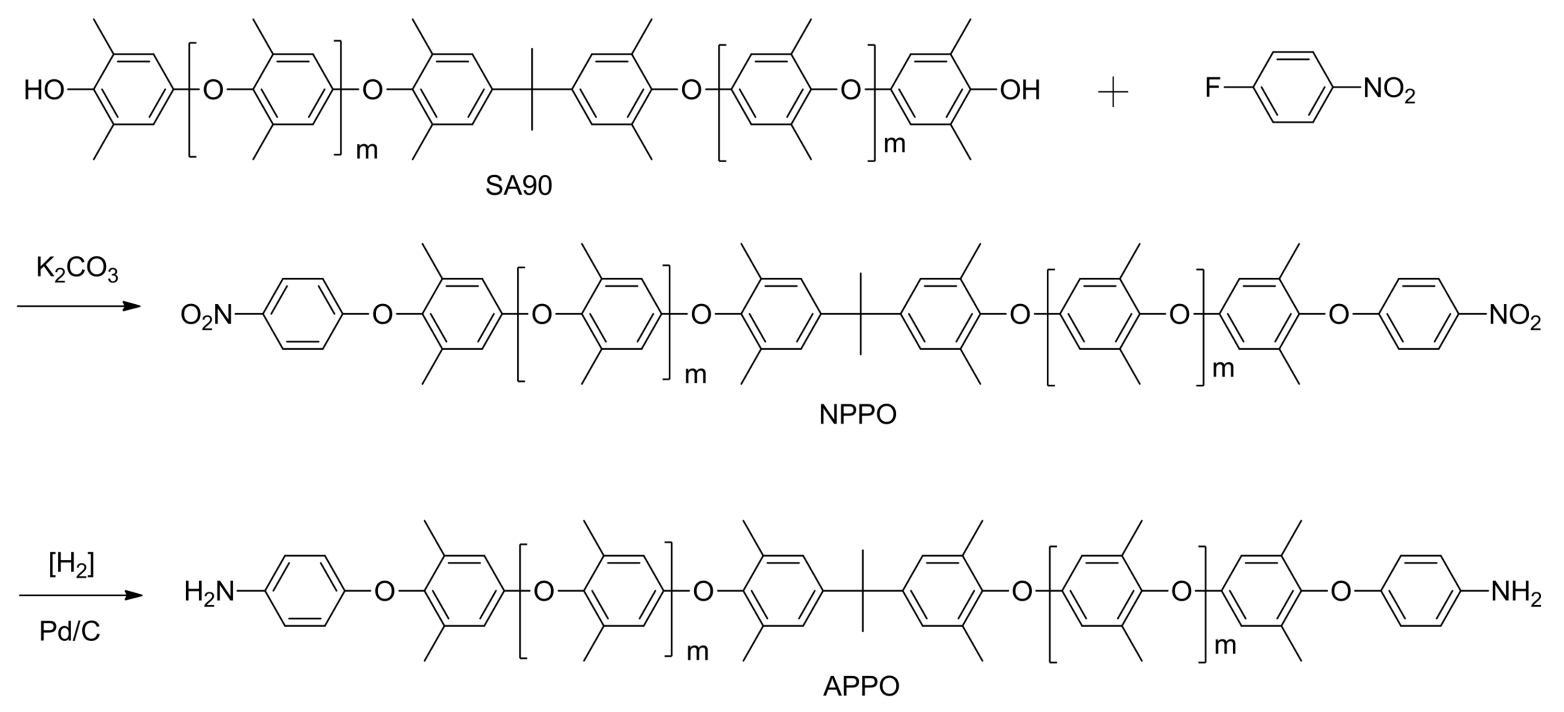
Syntheis of NPPO and APPO.

SA90 10.0 g (6.2 mmol), 4-fluoro-1-nitrobenzene 2.62 g (18.6 mmol), K_2_CO_3_ 2.57 g (18.6 mmol), and DMAc 50 mL were introduced into a 250 mL round-bottom glass flask equipped with a nitrogen inlet, condenser, and a magnetic stirrer. The solution was stirred at 80 °C for 24 h (Scheme 1). After that, the solution was poured into methanol/water = 1/1, yielding green powder. After drying the powder at 70 °C, the isolated yield was 91%. 

### 2.4. Synthesis of Amine End-Capped Oligo (2,6-Dimethyl Phenylene Oxide) (APPO)

NPPO 10.0 g (6.2 mmol), Pd/C 0.04 g, and THF 40 mL were introduced into a 250 mL Parr reactor. The solution was stirred hydrogen atmosphere at 25 °C, 140 psi for 24 h (Scheme 1). After that, the solution was poured into methanol, yielding gray powder. After drying the powder at 70 °C, the isolated yield was 93%. 

### 2.5. Two-Pot Synthesis of Telechelic Oligomer-Type Benzoxazine (P-APPO-2)

**Scheme 2:**
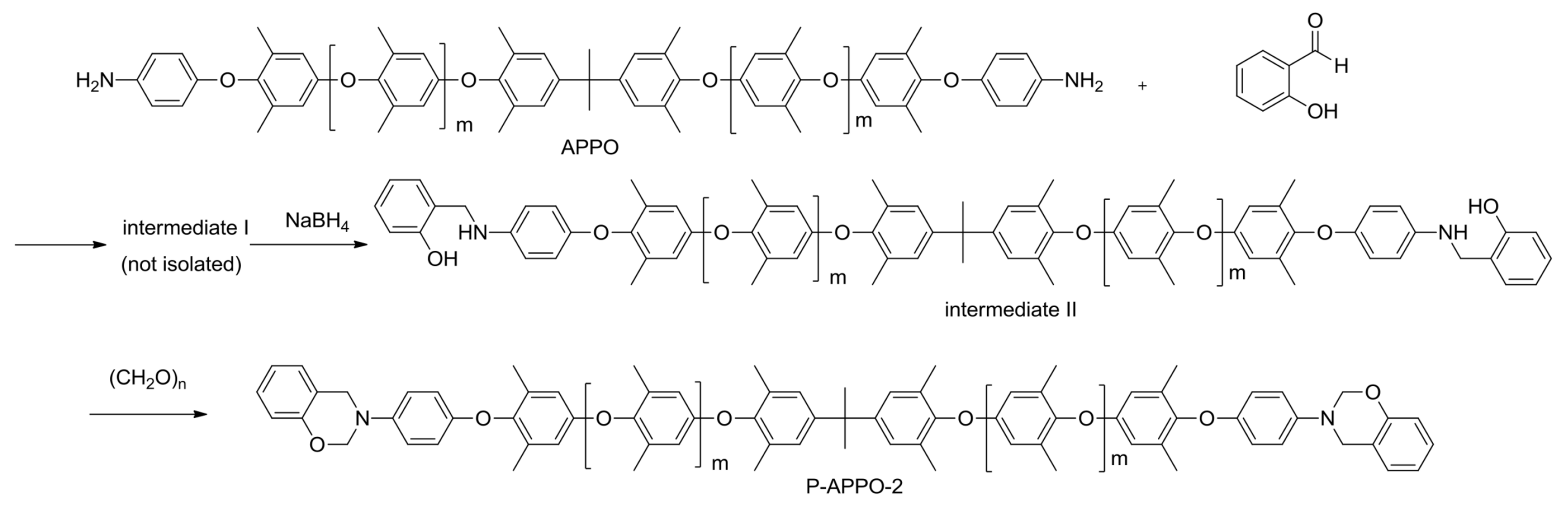
Two-pot synthesis of telechelic oligomer-type benzoxazine (P-APPO-2).

2-Hydroxybenzaldehyde 0.22 g (1.68 mmol), APPO 1.0 g (0.56 mol) and DMAc 10 mL were introduced into a 100-mL round bottom glass flask equipped with a nitrogen inlet and a magnetic stirrer. The reaction mixture was stirred at room temperature for 12 h. NaBH_4_ 0.066 g (1.68 mmol) was added and the solution was further stirred at room temperature for 24 h (Scheme 2). After that, the solution was poured into methanol/water = 1/1, yielding intermediate II with gray color. After drying the powder at 70 °C, the isolated yield was 93%. 

1.0 g (5.6 mmol) of intermediate II, paraformaldehyde 0.052 g (1.68 mmol), and toluene 5 mL were introduced into a 100-mL round bottom glass flask equipped with a condenser and a magnetic stirrer. The solution was stirred at 60 °C for 12 h. After that, the solution was poured into methanol, yielding a green powder. After drying the powder at 70 °C, the isolated yield was 93%. 

### 2.6. One-Pot Synthesis of Telechelic Oligomer-Type Benzoxazine (P-APPO-1)

**Scheme 3:**
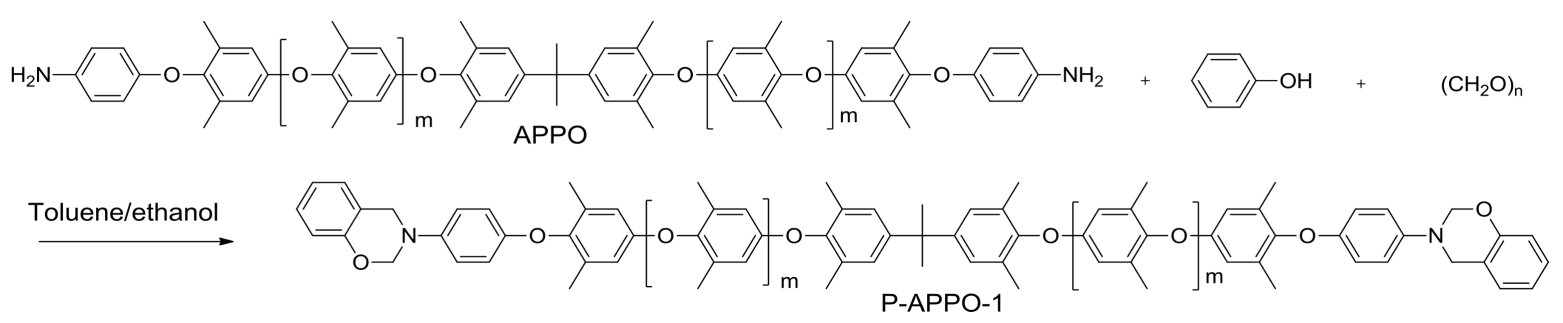
One-pot synthesis of telechelic oligomer-type benzoxazine (P-APPO-1).

APPO 2.0 g (0.56 mmol), phenol 0.32 g (1.68 mmol), paraformaldehyde 0.224 g (2.24 mmol), and toluène/ethanol (2/1) 30 mL were introduced into a 250 mL round bottom glass flask equipped with a condenser and a magnetic stirrer. The mixture was stirred at 80 °C for 24 h (Scheme 3). After that, the solution was poured into methanol, yielding a green powder. After drying the powder at 70 °C, the isolated yield was 91%. 

### 2.7. Syntheis of Main-Chain Type Benzoxazine Polymer (BPA-APPO)

**Scheme 4:**
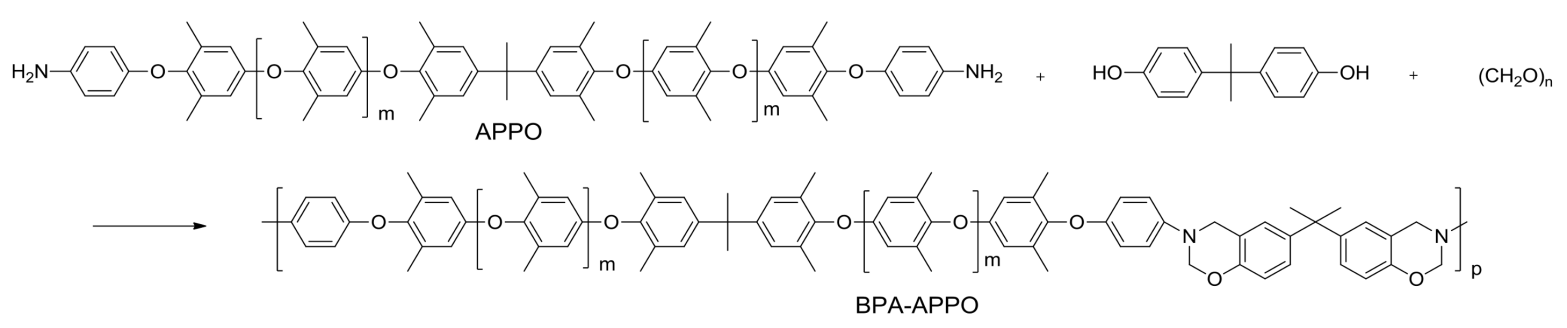
Synthesis of main-chain type benzoxazine polymer (BPA-APPO).

APPO 2.0 g (0.56 mmol), biphenol A 0.26 g (0.56 mmol), paraformaldehyde 0.14 g (2.24 mmol), and toluene/ethanol (2/1) 22 mL were introduced into a 250 mL round bottom glass flask equipped with a condenser and a magnetic stirrer. The mixture was stirred at 80 °C for 24 h (Scheme 4). After that, the solution was poured into methanol, yielding a gray powder. After drying the powder at 70 °C, the isolated yield was 93%. 

### 2.8. Sample Preparation and Curing Procedure

#### 2.8.1. The Self-Curing of P-APPO-2, P-APPO-1, and BPA-APPO

P-APPO-2, P-APPO-1, and BPA-APPO were thermally self-curing. Each benzoxazine was dissolved in xylene to make a solution with 20 wt % solid content. The solution was cast on a glass with an automatic film applicator. The resulting thin films were dried at 80 °C for 12 h. Then, cured by the program of 180 °C (2 h), 200 °C (2 h), and 220 °C (2 h) under a nitrogen atmosphere. The thermosets are named C-P-APPO-2, C-P-APPO-1, and C-BPA-APPO, respectively

#### 2.8.2. The Co-Curing of HP7200 with P-APPO-2, P-APPO-1, and BPA-APPO

P-APPO-2, P-APPO-1, and BPA-APPO were thermally co-curing with epoxy HP7200. The molar ratio of the oxazine moiety to oxirane (epoxy group) was controlled at one (The major curing reaction is the ring-opened phenolic OHs react with epoxy. The lone-pair electrons of nitrogen in oxazine act as the catalyst). Each benzoxazine and HP7200 were dissolved in xylene to make a solution with 20 wt % solid content. The solution was cast on a glass with an automatic film applicator. The resulting thin films were dried at 80 °C for 12 h. Then, cured by the program of 180 °C (2 h), 200 °C (2 h), and 220 °C (2 h) under a nitrogen atmosphere. The thermosets are named E-P-APPO-2, E-P-APPO-1, and E-BPA-APPO, respectively

#### 2.8.3. The Co-Curing of HP7200 with SA90

For property comparison, SA90 was thermally co-curing with epoxy HP7200. The molar ratio of the phenolic OH moiety to oxirane (epoxy group) was controlled at one. SA90 and HP7200 were dissolved in xylene to make a solution with 20 wt % solid content. The solution was cast on a glass with an automatic film applicator. The resulting thin film was dried at 80 °C for 12 h. Then, cured by the program of 180 °C (2 h), 200 °C (2 h), and 220 °C (2 h) under a nitrogen atmosphere. The thermoset is named E-SA90.

## 3. Results and Discussion

### 3.1. Synthesis and Characterization of APPO

**Figure 2:**
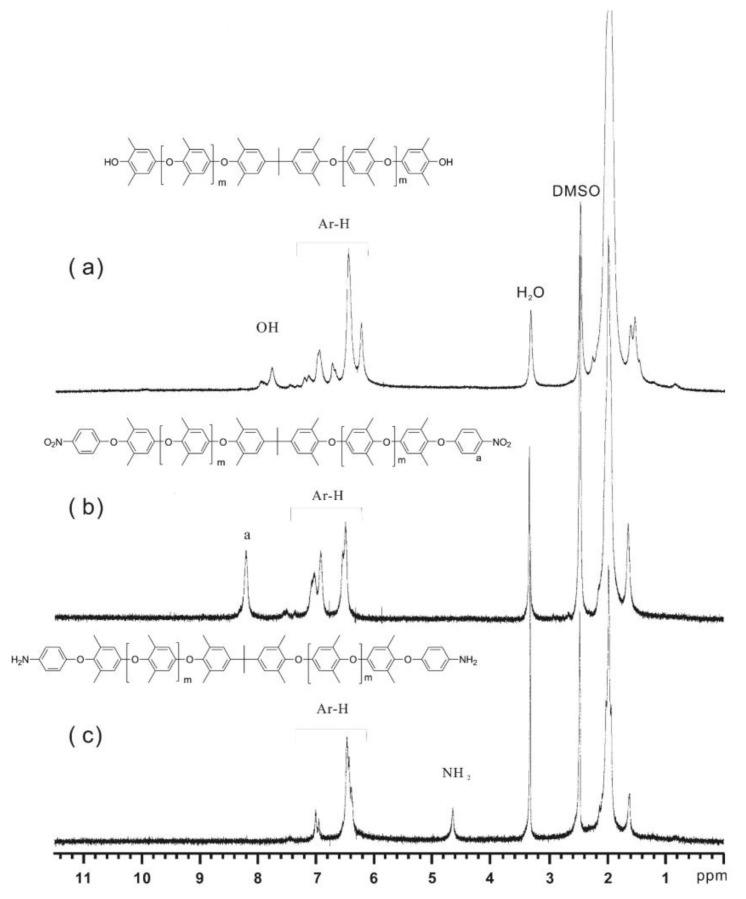
^1^H-NMR spectra of (**a**) SA90, (**b**) NPPO, and (**c**) APPO in DMSO-*d*_6_.

**Figure 3:**
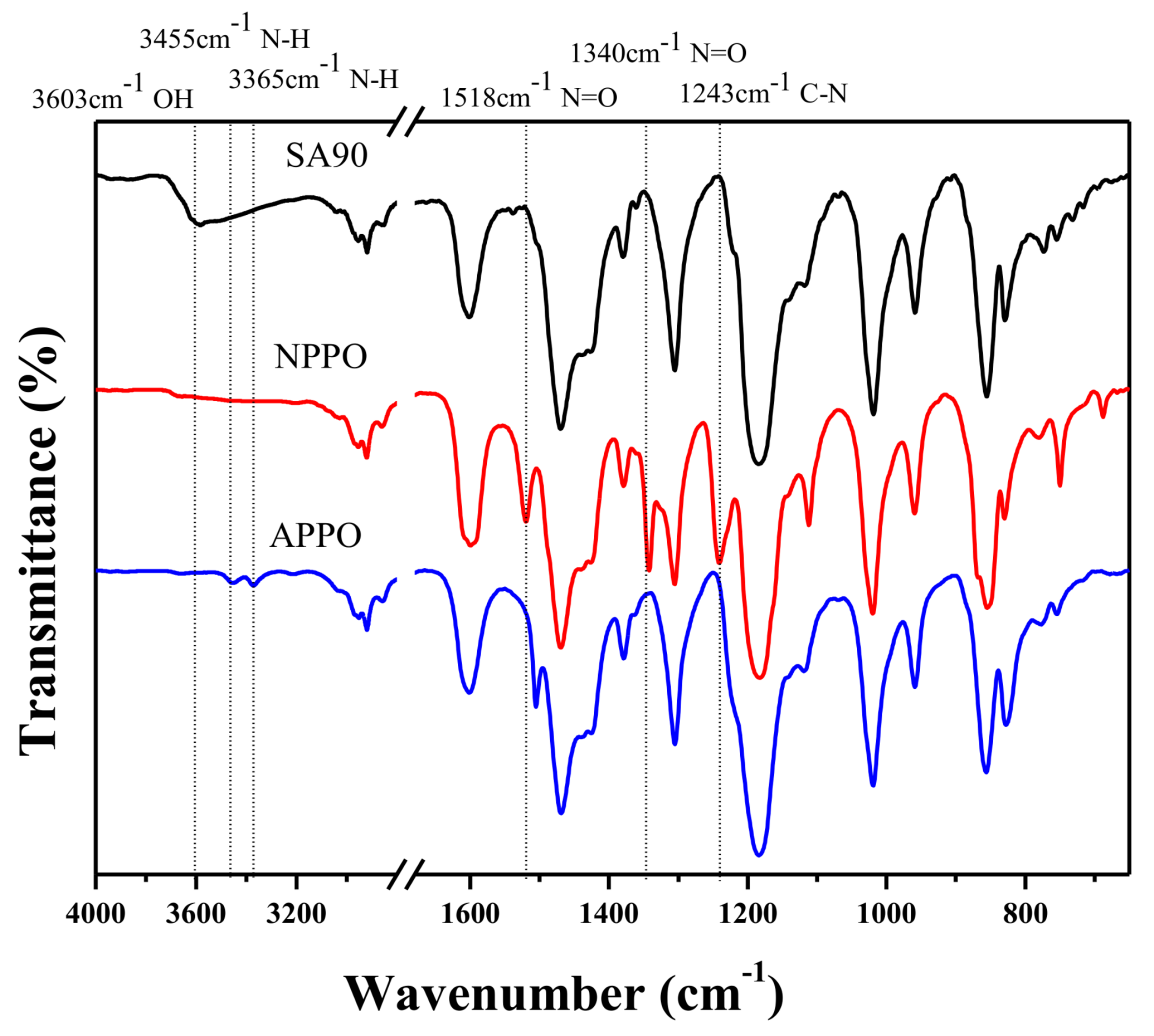
IR spectra of SA90, NPPO, and APPO.

APPO was prepared from the nucleophilic substitution of SA90 and fluoronitrobenzene, forming NPPO, and followed by catalytic hydrogenation of NPPO (Scheme 1). Figure 2 shows the ^1^H-NMR spectra of (a) SA90, (b) NPPO, and (c) APPO. The signals of phenolic OH at 7.8–8.0 ppm were observed in SA90. However, no phenolic OH signals were observed for NPPO, suggesting the completion of the reaction. The signals of Ar–H ortho to the nitro group were observed at 8.2 ppm for NPPO, while the signal disappeared in APPO. Furthermore, an amino peak at 4.6 ppm was clearly observed for APPO, indicating the successful hydrogenation. Figure 3 shows the IR spectra of SA90, NPPO, and APPO. The absorption of phenolic OH at around 3600 cm^−1^ was observed in SA90. However, no phenolic OH signals were observed for NPPO, suggesting the completion of the reaction. The absorptions of the nitro group were observed at 1340 and 1518 cm^−1^, while the signal disappeared in APPO. Furthermore, two amino absorptions at 3455 and 3365 cm^−^^1^ were clearly observed, indicating the successful hydrogenation.

### 3.2. Synthesis and Characterization of Benzoxazine P-APPO

**Figure 4:**
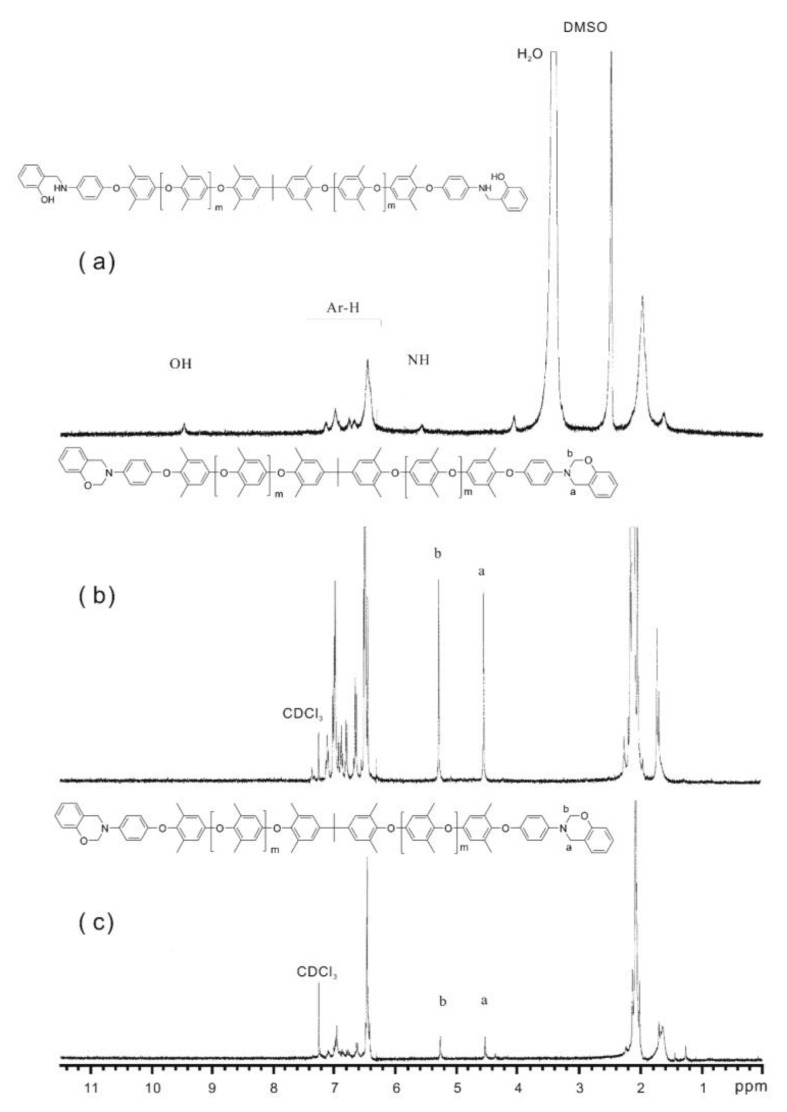
^1^H-NMR spectra of (**a**) intermediate II in DMSO-*d*_6_, (**b**) P-APPO-2, and (**c**) P-APPO-1 in CDCl_3_.

The phenol/APPO-based benzoxazine was prepared by two procedures in this work. The first approach is a two-pot procedure (Scheme 2) [38]. The procedure includes the reaction of 2-hydroxybenzaldehyde and APPO, yielding intermediate I (not isolated) with an o-hydroxy phenylimine linkage. The imine linkage of intermediate I was reduced by sodium borohydride, yielding intermediate II with a secondary amine structure. Then, paraformaldehyde was added to induce the ring closure of intermediate II, forming P-APPO-2. Figure 4 shows the ^1^H-NMR spectra of (a) intermediate II, and (b) P-APPO-2. A phenolic OH signal at 9.5 ppm and two new signals at 5.6 (NH) and 4.1 (CH_2_–NH) confirm the structure of intermediate II. The characteristic peaks of oxazine at 5.3 and 4.6 ppm confirm the formation of benzoxazine. No signal corresponding to N–CH_2_–Ph at around 3.80 ppm resulting from the ring-opened structure was observed, revealing the purity of synthesized P-APPO-2. The second approach is one-pot, Mannich condensation of phenol, APPO, and paraformaldehyde (Scheme 3). Using a traditional solvent such as dioxane leads to gelation. However, P-APPO-1 can be successfully prepared using a mixed solvent of toluene/ethanol, which is recommended in our previous work [39]. Figure 4c shows the ^1^H-NMR spectrum of P-APPO-1. The characteristic peaks of oxazine at 5.3 and 4.6 ppm confirm the formation of benzoxazines. However, a small unknown peak was observed at 4.3 ppm, indicating the purity is not as good as P-APPO-2. Generally, conventional biphenol-based benzoxazines often need extraction procedures to remove insoluble oligomer and unreacted monomers. However, no extraction or purification is required to prepare benzoxazines with moderate-to-high purity by these two approaches.

### 3.3. Synthesis and Characterization of Benzoxazine BPA-APPO

**Figure 5:**
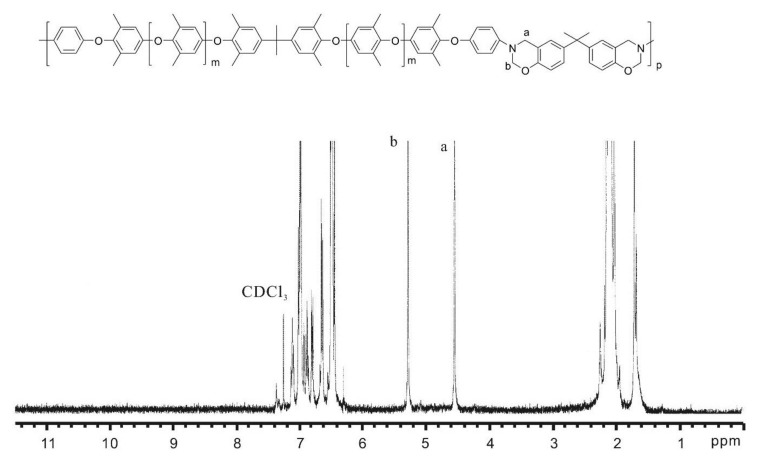
^1^H-NMR spectrum of BPA-APPO in CDCl_3_.

The main-chain type benzoxazine polymer (BPA-APPO) was prepared by one-pot, Mannich condensation of bisphenol A, APPO, and formaldehyde (Scheme 4) using toluene/ethanol as a solvent, which is recommended by our previous work [39]. Figure 5 shows the ^1^H-NMR spectrum of BPA-APPO. The characteristic peaks of oxazine at 5.2 and 4.5 ppm confirm the formation of benzoxazines. The number average molecular weight is 12,200 g/mol, and the weight average molecular weight is 19,800 g/mol. No signal corresponding to N–CH_2_–Ph at around 3.80 ppm resulting from the ring-opened structure was observed, revealing the purity of synthesized BPA-APPO.

### 3.4. Solubility

Table 1 lists the solubility data of SA90, P-APPO-1, P-APPO-2, and BPA-APPO. The benzoxazines show good solubility in various solvents except for DMSO. Especially, the main-chain type BPA-APPO also show the same solubility as telechelic oligomer-type P-APPO. The good solubility makes the benzoxazines readily for solution process in the copper clad laminate industry. 

### 3.5. DSC Thermograms

**Figure 6:**
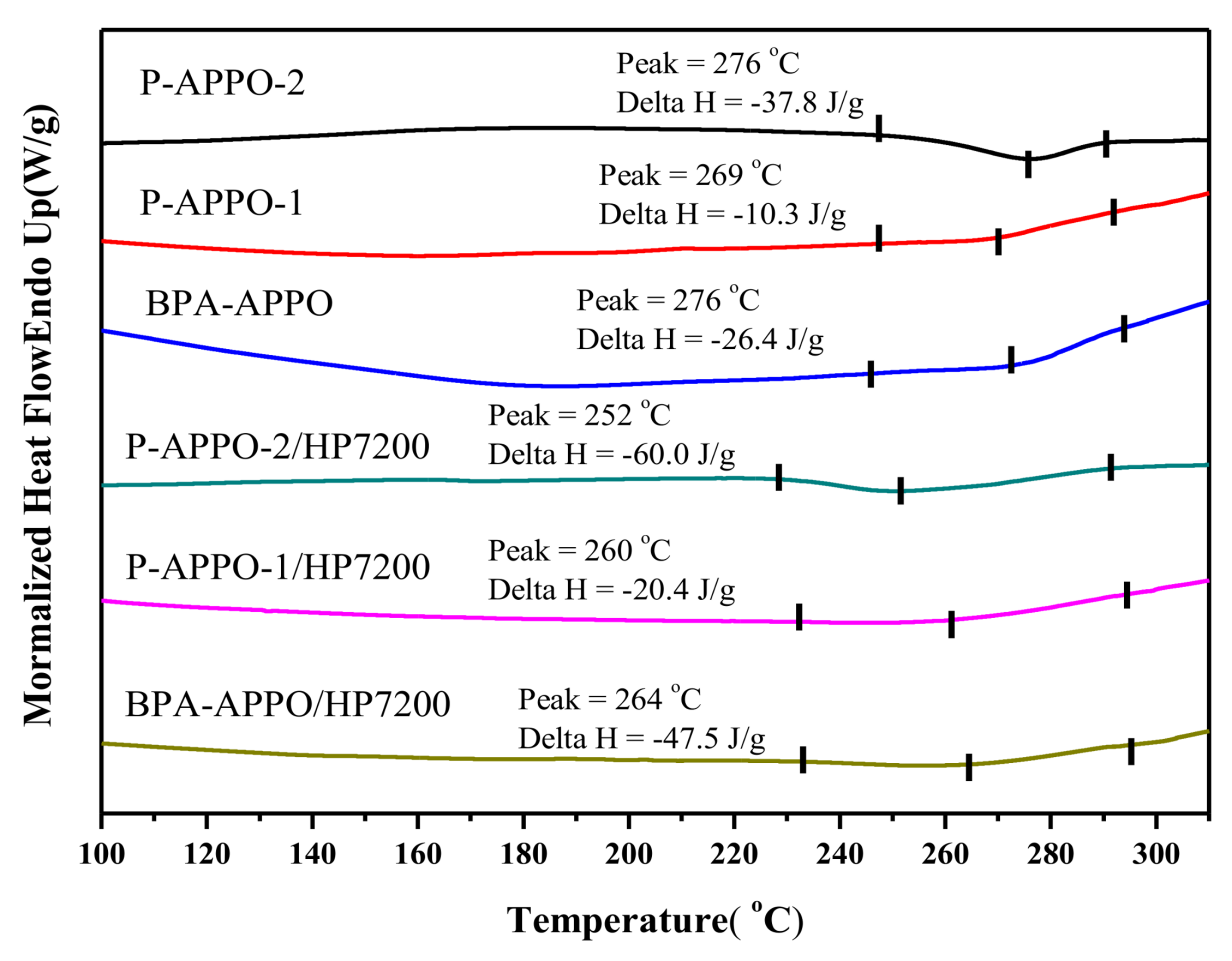
DSC thermograms of three PPO-containing benzoxazines and the benzoxazine/HP7200 blends.

Figure 6 shows the DSC thermograms of three PPO-containing benzoxazines and the benzoxazine/HP7200 blends. The exothermic peak temperature is around 270 °C for PPO-containing benzoxazines. The order of exothermic enthalpy is P-APPO-1 < BPA-APPO < P-APPO-2. The exothermic enthalpy of P-APPO-2 is more obvious than that of P-APPO-1, probably due to the higher purity of P-APPO-2 (Some ring-opened structures existed in the P-APPO-1, as supported by 4.3 ppm in Figure 4c, so the exothermic enthalpy of P-APPO-1 is smaller than P-APPO-2). BPA-APPO also displays relatively high purity, as supported by no ring-opened structure in Figure 5. However, the steric hindrance of polymer chains may hinder the access of benzoxazines, so its exothermic enthalpy is slightly lower than that of P-APPO-2. The exothermic peak temperature is around 260 °C for benzoxazine/HP7200 blend. In a single component system, it is well known that ring opening of oxazines leads to benzoxazine thermosets. However, in the benzoxazine/HP7200 blend system, the reaction routes become more complicated. According to the literature, there are several possible reaction routes might occur [40]. (1) The thermally-induced ring-opening polymerization of benzoxazines. (2) The homopolymerization of epoxy resin catalyzed by tertiary amine moiety in benzoxazine. (3) The co-reaction between epoxy and the phenolic OH resulting from ring-opening of benzoxazine. (4) The ring opening of benzoxazine catalyzed by the alkoxy anion, which is induced by the reaction between tertiary amine and epoxy moiety. This is probably the reason why the exothermic peaks of benzoxazine/HP7200 blend are slightly lower than those of neat benzoxazines. The order of exothermic enthalpy is P-APPO-1/HP7200 blend < BPA-APPO/HP7200 blend < P-APPO-2//HP7200 blend. The purity of benzoxazines and the mobility of molecules that determine the order of exothermic enthalpy in neat benzoxazine system explain the order of exothermic enthalpy in the benzoxazine/HP7200 system. 

### 3.6. Thermal Properties

**Figure 7:**
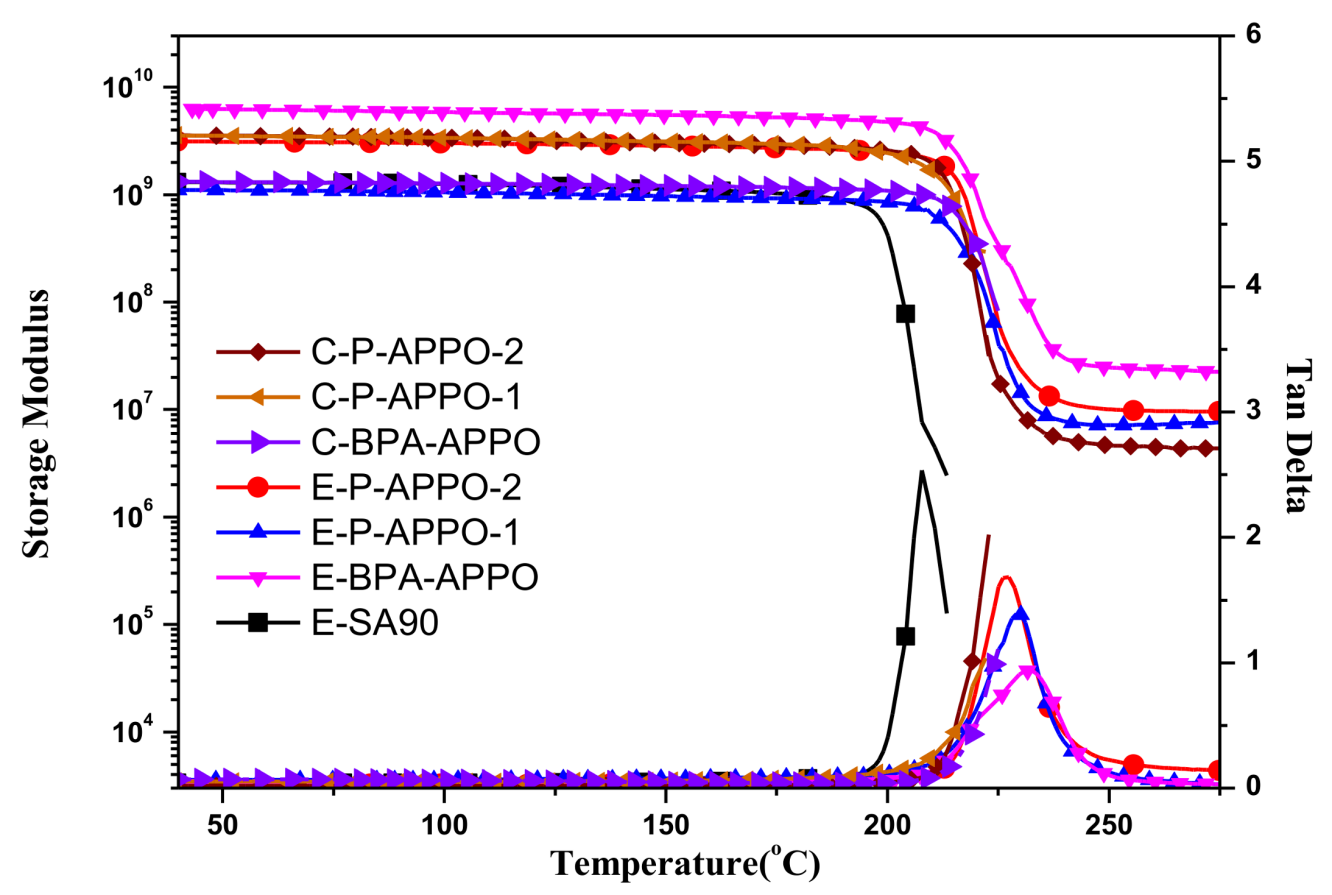
DMA thermograms of the prepared thermosets.

Figure 7 shows DMA thermograms of the prepared thermosets. The *T*_g_ data are listed in Table 2. The order of *T*_g_ value is C-BPA-APPO (225 °C for) > C-P-APPO-2 (222 °C) > C-P-APPO-1 (218 °C). 

In our previous work [41], we report the *T*_g_ value of a benzoxazine increases obviously with its molecular weight, but will level off after the molecular weight reaches a certain value. This explains the *T*_g_ value of C-BPA-APPO is only slightly higher than C-P-APPO since P-APPO is a telechelic oligomer benzoxazine, not a monomer-type benzoxazine. The higher *T*_g_ value of P-APPO-2 than that of P-APPO-1 is probably related to the larger exothermic enthalpy of P-APPO-2 that leads to a complete network. Generally, thermosets of bisphenol A/aniline-based poly(B-a), and bisphenol F/aniline-based poly(F-a) exhibit a *T*_g_ value at around 150–160 °C [1,3]. The values of 218 and 222 °C are relatively high compared with other benzoxazines, and demonstrate the high-*T*_g_ characteristic of PPO-containing benzoxazine thermosets. Table 2 also lists *T*_g_ data of the benzoxazine/epoxy thermosets. BPA-APPO/HP7200 (E-BPA-APPO) shows the highest *T*_g_ value of 232 °C while SA90/HP7200 (E-SA90) shows the lowest *T*_g_ (207 °C). E-P-APPO-2 and E-P-APPO-1 display higher *T*_g_ than E-SA90. The order of modulus of benzoxazine/HP7200 thermosets is E-BPA-APPO > E-P-APPO-2 > E-P-APPO-1 > E-SA90. The order of modulus is the same as that in *T*_g_. E-BPA-APPO shows higher modulus value while E-SA90 also shows the lowest modulus, demonstrating that our work is a successful approach to enhancing thermal properties.

**Figure 8:**
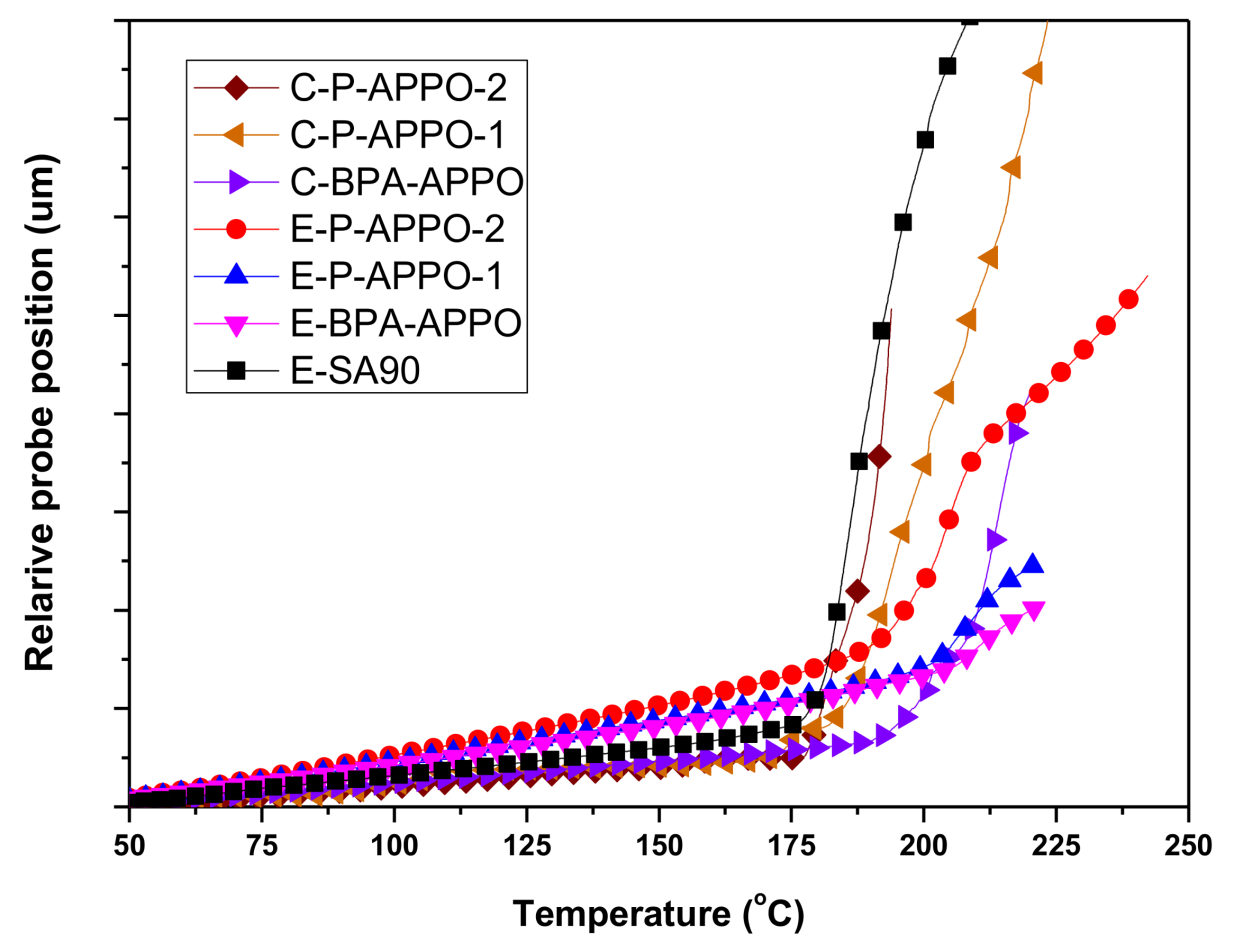
TMA thermograms of the prepared thermosets.

Figure 8 shows TMA thermograms of the prepared thermosets. The data of *T*_g_ and coefficient of thermal expansion (CTE) are listed in Table 2. The *T*_g_ values defined by TMA are 20–30 °C lower than those defined by DMA, but the trend is the same. The CTE values of thermosets of PPO-containing benzoxazines are around 56–60 ppm/°C, which are 10 ppm/°C lower than those of benzoxazine/HP7200 thermosets. 

Table 2 lists the thermal stability data of the prepared thermosets in a nitrogen atmosphere. The 5 wt % decomposition temperature is higher than 430 °C, and char yield at 800 °C is higher than 21%. Generally, the thermosets of bisphenol F/aniline-based and bisphenol A/aniline-based benzoxazines exhibit 5 wt % decomposition temperature at around 300–350 °C [1,3]. The result demonstrates the high thermal stability characteristic of the thermosets prepared in this work. 

### 3.7. Dielectric Properties

PPO is known for its moderate-to-low dielectric constant and very low dissipation factor. As a result, low-dissipation copper-clad laminated has prepared from SA9000 (a phenyl methacrylate-terminated PPO oligomer commercialized by SABIC) and OPE-2st (a vinyl benzyl ether-terminated PPO commercialized by MGC). To the best of our knowledge, no benzoxazine-terminated PPO (or PPO-containing benzoxazine) has been reported. Table 3 lists the dielectric properties of the prepared thermosets. All the thermosets show the same dielectric constant of 2.9 U, which is a moderate-to-low value compared with other thermosets (around 3.5 for generally epoxy thermosets [42,43,44,45,46]). In particular, they all show an extremely low dissipation factor. The dissipation factor is as low as 0.0040–0.0043 U for thermosets of PPO-containing benzoxazines, and 0.0050–0.0053 for benzoxazine/HP7200 thermosets. The dissipation factor is as good as that (0.005 U) of thermosets of commercialized OPE-2st by MGC and SA9000 by SABIC [47]. 

## 4. Conclusions

There are no free ortho positions for Mannich condensation in the phenolic hydroxyl-end capped oligo (2,6-dimethyl phenylene oxide), making it impossible to perform Mannich condensation directly. To the best of our knowledge, no PPO-containing benzoxazine has been reported before. To prepare the PPO-containing benzoxazine, we first transformed the phenolic hydroxyls to amino groups by a two-step procedure to obtain the amine end-capped oligo (2,6-dimethyl phenylene oxide) (APPO). Based on APPO, a telechelic oligomer-type benzoxazine and a main-chain type benzoxazine polymer (BPA-APPO) were prepared. The telechelic oligomer-type benzoxazine was prepared by a two-pot procedure (for P-APPO-2) and a one-pot procedure (for P-APPO-1). The former has higher purity than the latter, according to NMR and DSC data. Thermosets cured from BPA-APPO has the highest *T*_g_ (225 °C for C-BPA-APPO). The thermoset of P-APPO-2 show slightly higher *T*_g_ and lower *D*_f_ than that of P-APPO-1, indicating the purity of benzoxazine has an influence on thermal and dielectric properties. BPA-APPO/HP7200 (E-BPA-APPO) shows the highest *T*_g_ value of 232 °C while SA90/HP7200 (E-SA90) shows the lowest *T*_g_ (207 °C). E-P-APPO-2 and E-P-APPO-1 display higher *T*_g_ and lower *D*_f_ than E-SA90, demonstrating that our approach is a successful modification in simultaneously enhancing the thermal and dielectric properties. The 5 wt% decomposition temperatures of these thermosets are higher than 430 °C, and char yields at 800 °C are higher than 21%. The dissipation factor is as low as 0.0040–0.0043 U for thermosets of PPO-containing benzoxazines, and 0.0050–0.0053 for benzoxazine/HP7200 thermosets. The dissipation factor is as good as that (0.005 U) of thermosets of commercialized OPE-2st by MGC and SA9000 by SABIC. The combination of high *T*_g_ characteristic, high *T*_d_ characteristic, moderate-to-low *D*_k_, and extremely low dissipation factor makes the PPO-containing benzoxazine attractive for printed circuit boards for applications in high-frequency communication. 

## Figures and Tables

**Table 1:** Solubility data of SA90, P-APPO-1, P-APPO-2, and BPA-APPO.

Sample ^a^	NMP	DMSO	DMAc	DMF	MEK	THF	M-Cresol	Xylene	Toluene	CH_2_Cl_2_	CHCl_3_
SA90	++ ^b^	+−	++	++	++	++	+h	++	++	++	++
P-APPO-2	++	−	++	++	++	++	+h	++	++	++	++
P-APPO-1	++	−	++	++	++	++	+h	++	++	++	++
BPA-APPO	++	−	++	++	++	++	+h	++	++	++	++

^a^ solubility was tested with a 5 mg in 1 mL of solvent. ^b^ ++, soluble at room temperature; +h, soluble on heating; +−, partially soluble on heating; −, insoluble on heating.

**Table 2:** Thermal properties of the resulting polybenzoxazine.

Sample ID	*T*_g_ (°C) (DMA) ^a^	*T*_g_ (°C) (TMA) ^b^	CTE (ppm/°C) ^c^	*T*_d5%_ (°C) ^d^	CR (%) ^e^
C-P-APPO-2	222	194	60	443	28
C-P-APPO-1	218	185	56	442	28
C-BPA-APPO	225	197	60	437	28
E-P-APPO-2	230	195	71	435	22
E-P-APPO-1	227	185	75	430	22
E-BPA-APPO	232	203	66	433	21
E-SA90	207	174	70	416	22

^a^ Measured by DMA at heating rate of 5 °C/min; *T*_g_ value is determined from a peak temperature of the tan δ curve. ^b^ Measured by TMA at heating rate of 5 °C/min; *T*_g_ value is determined from onset temperature. ^c^ Coefficient of thermal expansion, recorded at 50 to 150 °C. ^d^ Temperatures corresponding to 5% weight loss by thermogravimetry at a heating rate of 20 °C /min in nitrogen. ^e^ Residual weight % at 800 °C in nitrogen.

**Table 3:** Dielectric properties of the prepared thermosets.

Sample	D_k_ (1 G Hz)	D_f_ (1 G Hz)
C-P-APPO-2	2.9 ± 0.01	0.0040 ± 0.00002
C-P-APPO-1	2.9 ± 0.02	0.0043 ± 0.00006
C-BPA-APPO	2.9 ± 0.05	0.0040 ± 0.00005
E-P-APPO-2	2.9 ± 0.05	0.0051 ± 0.00003
E-P-APPO-1	2.9 ± 0.02	0.0053 ± 0.00006
E-BPA-APPO	2.9 ± 0.03	0.0050 ± 0.00006
E-SA90	3.0 ± 0.02	0.0060 ± 0.00001
